# The Carbapenem Inactivation Method (CIM), a Simple and Low-Cost Alternative for the Carba NP Test to Assess Phenotypic Carbapenemase Activity in Gram-Negative Rods

**DOI:** 10.1371/journal.pone.0123690

**Published:** 2015-03-23

**Authors:** Kim van der Zwaluw, Angela de Haan, Gerlinde N. Pluister, Hester J. Bootsma, Albert J. de Neeling, Leo M. Schouls

**Affiliations:** Centre for Infectious Diseases Research, Diagnostics and Screening (IDS), National Institute for Public Health and the Environment (RIVM), Bilthoven, The Netherlands; Universitätsklinikum Hamburg-Eppendorf, GERMANY

## Abstract

A new phenotypic test, called the Carbapenem Inactivation Method (CIM), was developed to detect carbapenemase activity in Gram-negative rods within eight hours. This method showed high concordance with results obtained by PCR to detect genes coding for the carbapenemases KPC, NDM, OXA-48, VIM, IMP and OXA-23. It allows reliable detection of carbapenemase activity encoded by various genes in species of *Enterobacteriaceae* (e.g., *Klebsiella pneumoniae*, *Escherichia coli* and *Enterobacter cloacae*), but also in non-fermenters *Pseudomonas aeruginosa* and *Acinetobacter baumannii*. The CIM was shown to be a cost-effective and highly robust phenotypic screening method that can reliably detect carbapenemase activity.

## Introduction

The emergence and spread of carbapenemase-producing Gram-negative rods is a worldwide emerging public health threat [1–3]. Particularly in health care centers, this may pose a major problem as carbapenems are becoming more frequently needed to treat infections caused by Gram-negative bacteria that produce extended spectrum beta-lactamases (ESBL) [4,5]. To prevent spread of carbapenemase producers, rapid detection of these bacteria has become imperative [6]. Resistance to carbapenems is assessed in phenotypic susceptibility assays either on agar plates or in automated microbiology systems. However, high or low minimal inhibitory concentrations (MICs) do not necessarily reflect the production of carbapenemases, as other mechanisms such as porin loss or increased efflux pump activity, due to alterations in chromosomally located genes, can also cause resistance [7,8]. Since carbapenemase-encoding genes are often located on plasmids, this type of resistance is much more likely to spread [9]. Therefore, distinction between carbapenem-resistance mediated by carbapenemases and resistance mediated by other mechanisms is important for infection control. To identify possible carbapenemase involvement, PCRs targeting the genes encoding carbapenemases are often employed [10–12]. These methods can only detect known carbapenemase encoding genes and the number of carbapenemase encoding genes and allelic variants thereof is expanding rapidly. In contrast, a phenotypic assay may detect carbapenemase activity irrespective of the carbapenemase encoding gene sequence. In 2012, Nordmann et al. reported a new phenotypic test to detect the capability of an isolate to hydrolyse imipenem, the Carba NP test [13,14]. Even though this method is a huge improvement over methods such as the Modified Hodge Test, the costs are relatively high and some groups have reported difficulties with the Carba NP test, especially with mucoid isolates or isolates with weak carbapenemases such as OXA-48 [15]. In these cases only a subtle color change is observed which can easily be missed. This triggered us to develop an alternative bioassay designated as the Carbapenem Inactivation Method (CIM).

## Materials and Methods

### Strains and culture media

For validation of the CIM, a selection of 30 Gram-negative isolates was used. This selection included isolates obtained from different institutes across the world carrying known carbapenemase encoding genes and carbapenem susceptible isolates, according to the submitter (Table 1). In addition, 694 isolates submitted to the National Institute for Public Health and the Environment for the national surveillance of carbapenemase-producing *Enterobacteriaceae* (CPE) by Dutch medical microbiology laboratories (MMLs) during the first six months of 2012 and the first six months of 2013 were used. For the national surveillance of CPE in the Netherlands, Dutch MMLs are requested to submit *Enterobacteriaceae* isolates with an MIC for meropenem > 0.25 μg/ml. However, more than half of the isolates (411/694, 59%) sent in for CPE surveillance were non-fermenting Gram-negatives belonging to the genera *Pseudomonas* and *Acinetobacter*. Furthermore, 35% of the isolates had MICs below 0.25 μg/ml. Nevertheless, all isolates were included in this study.

**Table 1:** Isolates used for validation of the CIM.

Species	carbapenemase gene	CIM	CarbaNP
*Klebsiella pneumoniae* ^*1*^	KPC-2	+	+
*Klebsiella pneumoniae* ^*1*^	NDM-1	+	+
*Klebsiella pneumoniae* ^*1*^	OXA-48	+	+
*Klebsiella pneumonia*	OXA-48	+	+
*Klebsiella pneumonia*		-	-
*Klebsiella pneumonia*		-	-
*Klebsiella pneumonia*		-	-
*Escherichia coli*		-	-
*Escherichia coli*		-	-
*Escherichia coli*		-	-
*Escherichia coli*		-	-
*Escherichia coli*		-	-
*Escherichia coli* ATCC25922^*1*^		-	-
*Enterobacter cloacae*		-	-
*Enterobacter cloacae*		-	-
*Salmonella* Bareilly		-	-
*Salmonella* Heidelberg		-	-
*Pseudomonas aeruginosa* ^*1*^	VIM-2	+	+
*Pseudomonas aeruginosa*	VIM-2	+	+
*Pseudomonas aeruginosa* ^*1*^	IMP-1	+	+
*Pseudomonas aeruginosa* ^*1*^	GIM-1	+	+
*Pseudomonas aeruginosa* ^*1*^	SPM-1	+	+
*Pseudomonas aeruginosa*	AIM-1	+	+
*Pseudomonas fluorescens*	BIC-1	+	-
*Pseudomonas stutzeri* ^*1*^	DIM-1	+	+
*Acinetobacter baumannii*	OXA-23	+	+
*Acinetobacter baumannii*	OXA-40	+	+
*Acinetobacter baumannii*	OXA-58	+	+
*Acinetobacter baumannii*	OXA-143	+	+
*Acinetobacter baumannii* ^*1*^	SIM-1	+	+

^1^subset used for testing robustness

The species identification, as performed by the MMLs, was confirmed using MALDI-TOF (Bruker Daltonics GmbH, Bremen, Germany) and the MIC for all isolates was confirmed by E-test (BioMerieux Inc., Marcy L’Etoile, France). Culturing of isolates was done on Columbia Sheep Blood (bioTRADING Benelux BV, Mijdrecht, The Netherlands) and Mueller-Hinton agarplates (Oxoid Ltd, Hampshire, United Kingdom). An overview of all CPE surveillance isolates and their characteristics is displayed in Tables 2 and 3.

**Table 2:** Characteristics of *Enterobacteriaceae* isolates submitted for CPE surveillance during the first halves of 2012 and 2013.

				MIC >0.25 μg/ml			MIC ≤0.25 μg/ml	
*Enterobacteriaceae* species (n)	PCR	N	%	n	CIM+		n	CIM+
*Klebsiella pneumoniae* (88)	IMP	2	2.3	2	2			
	KPC	5	5.7	5	5			
	NDM	8	9.1	8	8			
	OXA-48	26	29.5	25	25		1	1
	VIM	1	1.1	1	1			
	pos.	42	47.7	41	41		1	1
	neg.	46	52.3	24	2		22	0
*Escherichia coli* (43)	IMP							
	KPC							
	NDM	7	16.3	7	7			
	OXA-48	13	30.2	12	12		1	1
	VIM	1	2.3	1	1			
	pos.	21	48.8	20	20		1	1
	neg.	22	51.2	12	1		10	0
*Enterobacter cloacae*(72)	IMP							
	KPC							
	NDM							
	OXA-48	3	4.2	3	3			
	VIM	1	1.4	1	1			
	pos.	4	5.6	4	4			
	neg.	68	94.4	48	0		20	0
Other *Enterobacteriaceae*(80)	IMP							
	KPC							
	NDM	1	1.3	1	1			
	OXA-48							
	VIM	2	2.5	1	1		1	1
	pos.	3	3.8	2	2		1	1
	neg.	77	96.3	17	0		60	0
All *Enterobacteriaceae* (283)	IMP	2	0.7	2	2			
	KPC	5	1.8	5	5			
	NDM	16	5.7	16	16			
	OXA-48	42	14.8	40	40		2	2
	VIM	5	1.8	4	4		1	1
	pos.	70	24.7	67	67		3	3
	neg.	213	75.3	101	3		112	0

N, total number of isolates

n, number of isolates within the MIC-category

**Table 3:** Characteristics of non-fermenter isolates submitted for CPE during the first halves of 2012 and 2013.

				MIC >0.25 μg/ml			MIC ≤0.25 μg/ml	
Non-fermenter species (n)	PCR	N	%	n	CIM+		n	CIM+
*Pseudomonas aeruginosa* (320)	IMP	4	1.3	4	4			
	KPC							
	NDM							
	OXA-48							
	VIM	43	13.4	43	43			
	pos.	47	14.7	47	47			
	neg.	273	85.3	268	1		5	0
Other *Pseudomonas* spp. (67)	IMP	2	3.0	2	2			
	KPC							
	NDM							
	OXA-48							
	VIM	6	9.0	6	6			
	pos.	8	11.9	8	8			
	neg.	59	88.1	58	5		1	0
*Acinetobacter baumannii* (18)	IMP							
	KPC							
	NDM	1	5.6	1	1			
	OXA-23*	12	66.7	12	10			
	OXA-48							
	VIM							
	pos.	12	66.7	12	10**			
	neg.	6	33.3	6	1			
Other *Acinetobacter* spp. (6)	IMP							
	KPC							
	NDM							
	OXA-23*							
	OXA-48							
	VIM							
	pos.							
	neg.	6	100.0	6	0			
All *non-fermenters* (411)	IMP	6	1.5	6	6			
	KPC							
	NDM	1	0.2	1	1			
	OXA-23*	12	2.4	12	10			
	OXA-48							
	VIM	49	11.9	49	49			
	pos.	67	16.3	67	65			
	neg.	344	83.7	338	7		6	0

N, total number of isolates

n, number of isolates within the MIC-category

* OXA-23 PCR was performed on *Acinetobacter* isolates only

** One *Acinetobacter baumannii* isolate was found positive for both NDM and OXA-23

### The Carbapenem Inactivation Method

**Fig 1:**
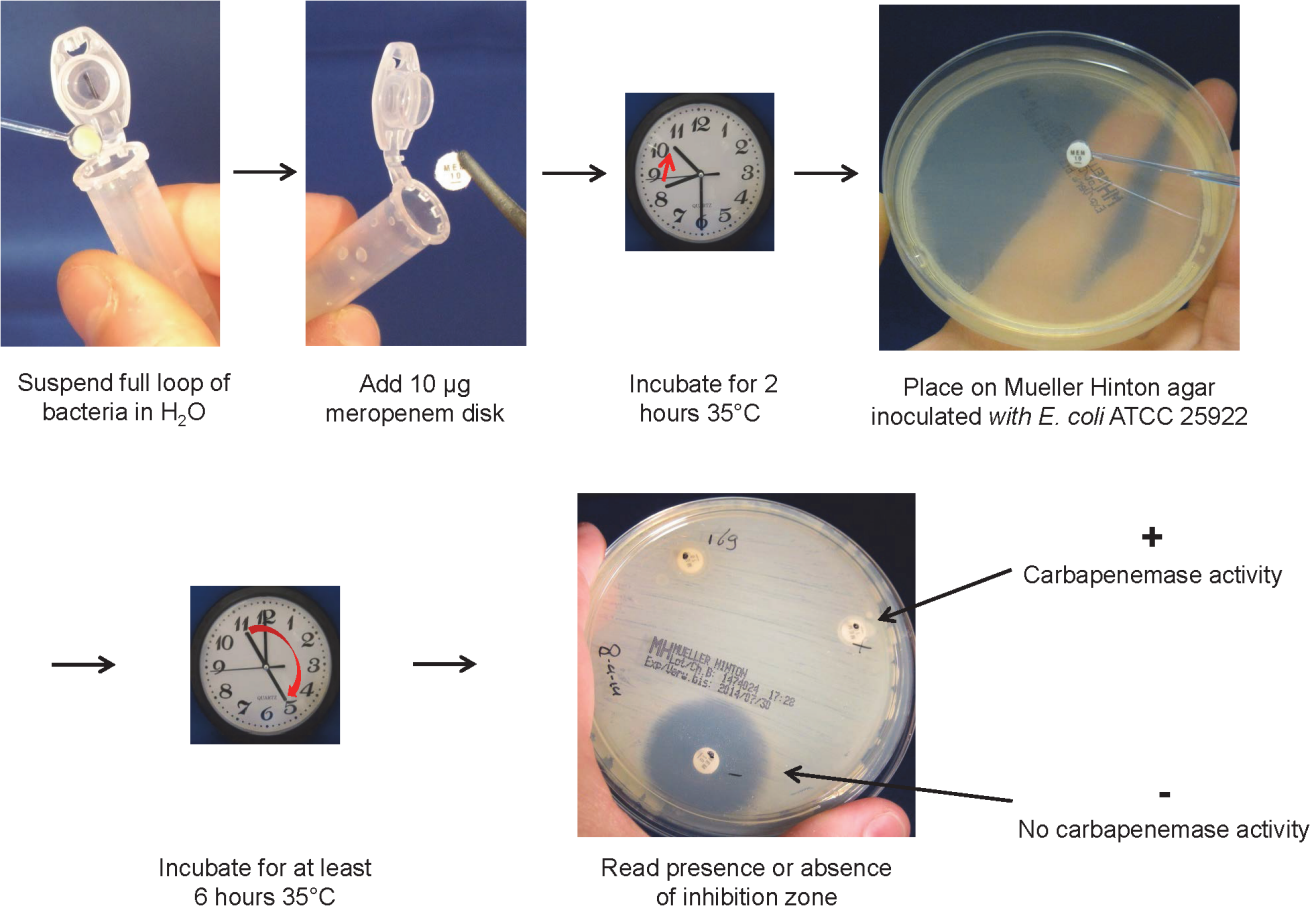
Schematic of the CIM.

To perform the CIM, a suspension was made by suspending a full 10 μl inoculation loop of culture, taken from a Mueller-Hinton or blood agar plate in 400 μl water. Subsequently, a susceptibility-testing disk containing 10 μg meropenem (Oxoid Ltd, Hampshire, United Kingdom) was immersed in the suspension and incubated for a minimum of two hours at 35°C. After incubation, the disk was removed from the suspension using an inoculation loop, placed on a Mueller-Hinton agar plate inoculated with a susceptible *E*. *coli* indicator strain (ATCC 29522) and subsequently incubated at 35°C. Inoculation of the Mueller-Hinton agar plate with the indicator strain was done with a suspension of OD_595_ 1.25 (correlates with a McFarland value of 0.5) streaked in three directions using a sterile cotton swab. If the bacterial isolate produced carbapenemase, the meropenem in the susceptibility disk was inactivated allowing uninhibited growth of the susceptible indicator strain. Disks incubated in suspensions that do not contain carbapenemases yielded a clear inhibition zone. If results are required within the same day, they can be read after six hours, but within the setting of our laboratory, we prefer reading results after overnight incubation (Fig. 1).

### PCR to detect the presence of carbapenemase encoding genes

A multiplex PCR that detects the genes encoding the predominant carbapenemases KPC, NDM, OXA-48like, VIM and IMP was used. To serve as an internal positive control, primers detecting the bacterial 23S rRNA gene were incorporated. This multiplex PCR was developed in-house and combines previously published (OXA-48L [10], IMP [11] and 23S [16]) and newly designed primers (KPC, NDM and VIM). For PCR, 12.5 μl of QiaGen Multiplex mix (QiaGen GmbH, Hilden, Germany) was mixed with 2 pmol of forward and reverse primers for KPC, OXA-48, VIM and 23S and with 6 pmol of the forward and reverse primers for NDM and IMP and supplemented with water to reach a final volume of 23 μl. Two μl of boilate from an isolate was added to the mix and 25 PCR cycles of 60 sec denaturation at 95°C, 60 sec annealing at 57.5°C and 60 sec elongation at 72°C were run. In addition, *Acinetobacter* isolates were tested for the presence of the gene encoding for OXA-23 in a separate PCR [12]. Finally, for isolates that demonstrated carbapenemase activity using the CIM, but did not yield a PCR product using the multiplex PCR described above, three additional PCRs were performed. These separate PCRs, targeting BIC [11] and additional variants of OXA-48L and IMP [17], were performed using 10 pmol of each primer and the same PCR conditions as the multiplex PCR.

The primer sequences and the expected PCR product sizes are listed in Table 4.

**Table 4:** Primers used for PCR detection of carbapenemase encoding genes.

Primer name	Primer sequence	Product size (bp)	Reference
KPC_mp-F	CTTGTCTCTCATGGCCGCTGG	449	this study
KPC_mp-R	ACGGAACGTGGTATCGCCGAT		this study
NDM_mp-F	TCCTTGATCAGGCAGCCACC	591	this study
NDM_mp-R	CGCATTAGCCGCTGCATTGA		this study
VIM_mp-F	GCMCTTCTCGCGGAGATTGA	257	this study
VIM_mp-R	TGCGCAGCACCRGGATAGA		this study
OXA48L-F	GCTTGATCGCCCTCGATT	281	[10]
OXA48L-R	GATTTGCTCCGTGGCCGAAA		[10]
IMP-F	GGAATAGAGTGGCTTAAYTCTC	233	[11]
IMP-R2	GGTTTAAYAAAACAACCACC		[11]
23S_mp-F	GCGATTTCYGAAYGGGGRAACCC	371*	[16]
23S_mp-R	TTCGCCTTTCCCTCACGGTACT		[16]
OXA_23-F	GATCGGATTGGAGAACCAGA	501	[12]
OXA_23-R	ATTTCTGACCGCATTTCCAT		[12]
BIC-F	TATGCAGCTCCTTTAAGGGC	537	[11]
BIC-R	TCATTGGCGGTGCCGTACAC		[11]
OXA48L-R2	CCCTAAACCATCCGATGTGG	516	this study
IMP-mp-F	GGAATAGRGTGGCTTAATTC		[17]**
IMP-mp-R1	GACTTTGGCCAAGCTTYTA	285	[17]
IMP-mp-R2	GTAAGCTTCAAGAGCGACG	375	[17]

* product size for *E*. *coli*, size varies for different species

** Zhao and Hu 2011, F and R1 primers adapted from D-F and A-R primer, respectively; R2 primer identical to C-R primer.

### Next-generation sequencing

Four isolates with discrepant results for CIM in comparison to PCR, an *E*. *coli*, *K*. *pneumoniae*, *A*. *baumannii* and a *Proteus mirabilis* isolate, have been analyzed using next-generation sequencing.

DNA was extracted using the QuickExtract Bacterial DNA Extraction Kit (Epicentre, Madison, USA) according to the manufacturer’s instructions, followed by proteinase K (QiaGen GmbH, Hilden, Germany) digestion and precipitation. Library preparation and sequencing of bacterial genomes was performed by BaseClear (Leiden, the Netherlands) using the Illumina Nextera XT kit and the HiSeq 2500 with a paired-end 100 cycles protocol. For detection of beta-lactamase genes, a reference list of relevant genes was composed based on those listed on the Lahey Clinic website (http://www.lahey.org/Studies/). Trimmed sequence reads were mapped to this reference list, after which mapped reads were extracted, assembled into contigs and antibiotic resistance determinants were identified by BLAST analysis of the resulting contigs against the same reference list. Sequence reads were deposited to the European Nucleotide Archive (ENA) under project accession PRJEB8575 (http://www.ebi.ac.uk/ena/data/view/PRJEB8575).

### Carba NP and CarbAcineto NP

A selection of isolates has also been tested for carbapenemase activity with Carba NP and CarbAcineto NP using the most recently published protocols [18,19]. In short, a suspension of a fourth to a third of a 10 μl loop of culture in 100 μl of commercially available lysis buffer (B-PERII, Thermo Scientific Pierce, Rockford, USA) was mixed with 100 μl of a phenol red solution of pH 7.8 containing 0.6 mg imipenem monohydrate. If a carbapenemase is present in the solution, the hydrolysis of the imipenem will lower the pH, causing the phenol red to turn from red to orange or yellow. For *Acinetobacter*, this protocol has two modifications: the use of a 5 M NaCl solution instead of the B-PERII lysis buffer and a full 10 μl loop of culture as an inoculum.

## Results

### Validation of the CIM

To validate the robustness of the CIM, the influence of a number of variables on the outcome of the assay was determined. First, the required density of the bacterial suspension used to immerse the meropenem disk was assessed. For this purpose, we used three isolates: two isolates yielding an OXA-48 PCR product: a *K*. *pneumoniae* with an MIC of 32 μg/ml and an *E*. *cloacae* with an MIC of 1.5 μg/ml for meropenem and *E*. *coli* ATCC 25922 as a negative control. Bacterial suspensions with optical density OD_595_ 2.6, OD_595_ 13 (density obtained with a loop full of bacteria), and OD_595_ 65 were used (OD_595_ 2.6 correlates with a McFarland value of 11.3). The suspension with OD_595_ 2.6 yielded negative results for both OXA-48 positive isolates, whereas both OD_595_ 13 and OD_595_ 65 yielded positive CIM results. The CIM remained negative for all densities of *E*. *coli* ATCC 25922.

A subset of ten isolates from the selection in Table 1 was used to determine the influence of a number of other variables. First, the incubation time of the meropenem disk in the bacterial suspension was varied from 30 minutes to overnight. Results indicated that for this subset, 30 minutes were sufficient to obtain correct results. After overnight incubation, the results could still be assessed, although decreased inhibition zones for the negative control indicated partial auto-hydrolysis of the meropenem in the disk. However, this was only tested with a small subset to illustrate the robustness of the method. To improve detection in cases of low-level carbapenemase activity and to obtain an assay that can still be interpreted within one working day, a minimum incubation time of 2 hours was chosen. Incubation temperatures of 30°C, 35°C and 37°C all yielded identical results. Suspensions made from isolates grown on Mueller-Hinton agar and Columbia sheep blood agar plates both resulted in the correct detection of carbapenemase activity. However, in cultures grown on Oxoid CRE plates (Oxoid Ltd, Hampshire, United Kingdom), which contain a chromogenic substrate and a carbapenem, carbapenemase activity could not be detected in four out of nine carbapenemase positive isolates. Suspensions made from cultures that had been maintained refrigerated on agar plates for up to two weeks gave identical results as suspensions made from fresh cultures. Similarly, bacterial suspensions made from fresh cultures that were subsequently stored for up to two weeks at 4°C, retained their capability to inactivate the carbapenem in the disk. The CIM was performed using susceptibility-testing disks containing 10 μg meropenem from two different suppliers (Oxoid Ltd, Hampshire, United Kingdom and Mast Group Ltd, Merseyside, United Kingdom), which yielded identical results. The addition of zinc sulfate to the suspension, used to stimulate metallo-betalactamases such as NDM, was tested but no beneficial effect could be demonstrated. Finally, to assess the influence of human skill on the performance of the CIM, different laboratory staff tested the previously mentioned selection of 30 Gram-negative isolates (Table 1) and no discrepancies were found.

### Application of the CIM on surveillance isolates

Of the 283 *Enterobacteriaceae* included in the study, 70 (24.7%) were shown to carry a carbapenemase encoding gene by PCR and all PCR-positives (100%) were shown to produce carbapenemase by the CIM (Table 2). A *Proteus mirabilis* isolate with an MIC of 0.094 μg/ml for meropenem yielded a VIM PCR product, but was initially negative for CIM. Like all isolates for which PCR and CIM yielded discrepant results, the analysis was repeated and CIM was positive in the second analysis. Two other isolates with MICs ≤ 0.25 μg/ml (*K*. *pneumoniae* and *E*. *coli*) yielded an OXA-48 PCR product and both isolates were shown to produce carbapenemase. Three isolates (two *K*. *pneumoniae* and one *E*. *coli*) produced carbapenemase, but were PCR-negative using our multiplex PCR. However, they did yield an OXA-48-like PCR product using a modified reverse primer (OXA-48L-R2, Table 4). Sanger sequencing of the PCR product obtained with this modified primer revealed that all three had allele OXA-181, which cannot be detected with the original primer set. Nearly half of the *K*. *pneumoniae* (47.7%) and *E*. *coli* (48.8%) isolates included in this study yielded a PCR product representing a carbapenemase encoding gene and all these isolates were CIM-positive. However, we detected a carbapenemase encoding gene in only a minor fraction of the *E*. *cloacae* (5.6%) and the other *Enterobacteriaceae* (3.8%). Again, all PCR-positives were shown to produce carbapenemase by CIM. Of the 26 OXA-48 positive *K*. *pneumoniae* isolates, five had a mucoid colony morphology, but this did not affect the ability of CIM to detect carbapenemase activity. Forty *Enterobacteriaceae* isolates (14.1%) had MICs for meropenem that were above the EUCAST clinical breakpoint of resistance (>8 μg/ml). In 14 of these (35.0%) no carbapenemase encoding gene or carbapenemase activity was detected.

The collection contained a large number of non-fermenting Gram-negatives all belonging to the *Pseudomonas* and *Acinetobacter* genera (Table 3). Sixty-seven of the 411 isolates (16.3%) yielded a PCR product and 65 of these samples (97.0%) yielded a positive CIM. Only in two *A*. *baumannii* isolates carrying *bla*_OXA-23_, carbapenemase activity could not be detected with CIM. All 67 isolates had MICs >0.25 μg/ml. Among the *Pseudomonas* isolates, VIM was the most frequently detected carbapenemase encoding gene, present in 49 of the 387 isolates (12.7%). In *A*. *baumannii*, OXA-23 was the predominant carbapenemase encoding gene, detected in 12 of the 18 isolates (66.7%). None of the other *A*. spp. were PCR positive. Of the 344 PCR-negative non-fermenter isolates, only seven were CIM-positive (2.0%) and all of these isolates had MICs >0.25 μg/ml. Three of these isolates were found to contain a carbapenemase-encoding gene using additional PCRs; two *Pseudomonas* isolates were BIC positive [11] and one *Pseudomonas* isolate was found to carry an IMP variant not detected by our multiplex PCR) [17]. In addition, one CIM-positive *A*. *baumannii* isolate was found to contain an OXA-72 gene by next-generation sequencing. Compared to the *Enterobacteriaceae*, a larger portion of the non-fermenter isolates had MICs for meropenem that were above the EUCAST clinical breakpoint for resistance (222/411, 54.0%). Yet, 164 (73.8%) of these were negative in both PCR and CIM.

### Comparison of CIM with Carba NP

A selection (n = 116) of the isolates subjected to CIM has also been tested with Carba NP or CarbAcineto NP using the most recently published protocols [18,19]. These isolates include all isolates used for validation (Table 1), all OXA-48 positive *K*. *pneumoniae* isolates as well as all CIM/PCR discrepant isolates, supplemented with a random selection of other isolates used (Table 5). Four isolates (3.4%) gave different results for CIM than for Carba NP. Three of these were isolates found to contain a carbapenemase encoding gene (two *bla*_BIC_ and one *bla*_OXA-48like_) where CIM was positive and Carba NP was (false-) negative. The fourth isolate was a CIM positive *Pseudomonas* spp. for which no carbapenemase encoding gene was identified, but carbapenemase activity could not be confirmed with Carba NP.

**Table 5:** Comparison of the CIM and Carba NP test.

Species	PCR	n	CIM+	CarbaNP+
*Klebsiella pneumoniae*(38)	KPC	1	1	1
	NDM	5	5	5
	OXA-48	28	28	28
	IMP	2	2	2
	neg	2	0	0
*Escherichia coli* (8)	NDM	1	1	1
	OXA-48	3	3	2
	neg	4	0	0
*Enterobacter cloacae* (7)	OXA-48	2	2	2
	VIM	1	1	1
	neg	4	0	0
Other *Enterobacteriaceae*(16)	NDM	1	1	1
	VIM	1	1	1
	neg	14	0	0
*Pseudomonas aeruginosa*(5)	VIM	3	3	3
	IMP	1	1	1
	neg	1	0	0
*Pseudomonas* spp. (8)	VIM	1	1	1
	IMP	1	1	1
	BIC	2	2	1
	neg	4	2	1
*Acinetobacter baumannii*(3)	OXA-23	2	0	0
	OXA-72	1	1	1
*Acinetobacter* spp. (1)	neg	1	0	0
All isolates		86	54	51

## Discussion

The concept of demonstrating enzymatic hydrolysis of beta-lactam antibiotics by incubating them with bacterial suspensions is not new and dates back to the late 70s [20]. However, the CIM is the first to use antibiotic susceptibility-testing disks, which are globally available at low cost and have long shelf lives, as substrate aliquots for this. This greatly improves practicality and reduces costs and labor. Furthermore, this method was shown to be unaffected by changes in variables such as incubation temperature or time, disk manufacturer, laboratory staff and the age of the culture or bacterial suspension making it a cost-effective and highly robust phenotypic screening method that can reliably detect carbapenemase activity. As it requires no specialized equipment, reagents or skill and low hands-on time to perform, it is a high throughput method enabling screening of large numbers of isolates in most medical microbiological laboratories.

The application of the CIM in this study shows that it is capable of detecting carbapenemase production in Gram-negatives allowing distinction between carbapenem-resistance due to beta-lactamase activity and reduced permeability. There was a high concordance (100% for *Enterobacteriaceae* and 98,8% for non-fermenters) between PCRs that detect carbapenem encoding genes and carbapenemase activity detected by CIM. After application of additional PCRs to the discrepant isolates, all findings with CIM were in accordance with PCRs among *Enterobacteriaceae*, giving CIM a positive predictive value and a negative predictive value of 100% for *Enterobacteriaceae* compared to PCRs. One *P*. *mirabilis* isolate showed a remarkable result: it carried a *bla*_VIM-1_ gene, but had a low MIC for meropenem (0.094) and initially yielded a negative CIM. However, when repeated CIM was positive. Carba NP showed a weak positive reaction for this isolate. Preliminary results obtained by next-generation sequence analysis of this isolate strain showed that the bla_VIM-1_ gene itself was intact and located on a class I integron-like structure as has been described for other VIM-producing species, such as *P*. *aeruginosa*, *K*. *pneumoniae* and another *P*. *mirabilis* isolate [21–23]. Expression of resistance cassettes from integron 1-type structures is mediated by the P_ant_ promoter. Variations in P_ant_ promoter sequence have been observed in several naturally occurring integrons, and have been shown to differ at least 20-fold in strength [24], with the promoter sequence found in our *P*. *mirabilis* isolate (TGGACA-N17-TAAGCT) belonging to the weaker promoters. Weak expression of *bla*_VIM-1_ would result in low-level carbapenemase activity around the detection-threshold of CIM, explaining the discrepancy between the two assays performed with this isolate as well as the low MIC observed.

Initially, there were seven isolates among the non-fermenters that were CIM-positive, but for which no gene could be identified using the multiplex PCR. After applying additional PCRs, four isolates remained PCR-negative, one of which could be explained by an OXA-72 gene identified using next-generation sequencing. Of the remaining three false-positive isolates, two were also Carba NP positive, suggesting the presence of a carbapenemase currently undetectable by our genotypic methods. Conversely, for two OXA-23 positive *A*. *baumannii* isolates no phenotypic carbapenemase activity could be detected using CIM. When CIM was performed using an extended incubation time of four hours instead of two, CIM was positive for both *Acinetobacter* isolates, indicating very weak carbapenemase activity. Carbapenemase activity could also not be detected for these isolates using CarbAcineto NP (Table 5). The difficulties detecting low-level carbapenemase activity among both these *A*. *baumannii* isolates and the *P*. *mirabilis* isolate mentioned earlier can be regarded as a limitation of the CIM. Use of an extended incubation time of four hours for all *Acinetobacter* and *Proteus* isolates might overcome this limitation, but further research is required to determine if this will improve detection without compromising specificity. The five discrepant non-fermenter isolates are considered false-positive and false-negative. This leads to a positive predictive value of 96.3% and a negative predictive value of 99.4% for CIM among non-fermenters compared to PCRs.

Additionally, many isolates apparently were resistant for meropenem, but were negative in both PCR and CIM, suggesting that carbapenem-resistance was mediated by other mechanisms than carbapenemase activity in these isolates, which is currently being investigated by next-generation sequencing.

In comparison to the Carba NP method [13,14,18,19], CIM has similar high performance (96.6% concordance, 112/116), but significantly lower cost (€0.60 compared to €13 in our setting). Prices mentioned are based on imipenem and B-PERII buffer as reagents for CarbaNP and a meropenem disk, Mueller-Hinton agar plate and sterile cotton swab required for CIM. In addition, we believe that CIM is even easier to perform than Carba NP since there is no need to prepare a fresh pH adjusted imipenem solution for each test. Moreover, in our experience, interpretation of results using CIM is also much easier compared to the Carba NP test. Some of the isolates tested with Carba NP showed a very weak color change that could easily have been missed in a routine screening setting. However, we should emphasize that this study was not intended to compare CIM with Carba NP.

Another alternative to detect carbapenemase activity among isolates is an assay based on matrix-assisted laser desorption ionization time-of-flight mass spectrometry (MALDI-TOF MS). High performance has been reported for this technique, but none of these studies feature strain collections as large as the one described here [25–28]. Furthermore, this technique requires expensive specialized equipment, which may not be present in all medical microbiological laboratories, making it less suited for screening purposes.

The CIM concept has the potential to also be applied to assess enzymatic hydrolysis of other antibiotics, e.g. allowing detection of ESBL activity. Preliminary experiments in our laboratory have shown this may be feasible. This will need to be further investigated, but it could represent an additional advantage over Carba NP, which can only be applied to antibiotics that generate sufficient acidity upon hydrolysis.

In conclusion, we have shown that the CIM provides a low cost alternative for the Carba NP test. It allows easy and rapid identification of carbapenemase activity. This provides a tool to identify carbapenem-resistant Gram-negative bacteria that may easily spread in health care settings.
